# A61 TARGETING A GUANINE QUADRUPLEX IN THE HEPATITIS B VIRUS COVALENTLY CLOSED CIRCULAR DNA GENOME USING SINGLE DOMAIN ANTIBODIES

**DOI:** 10.1093/jcag/gwac036.061

**Published:** 2023-03-07

**Authors:** S D'souza, G Balderas Figueroa, M D Badmalia, T R Patel, C Coffin

**Affiliations:** 1 Microbiology, Immunology, and Infectious Diseases, University of Calgary Cumming School of Medicine, Calgary; 2 Chemistry and Biochemistry, University of Lethbridge, Lethbridge; 3 Discovery Lab Faculty of Medicine and Dentistry, University of Alberta, Edmonton; 4 Department of Medicine, University of Calgary Cumming School of Medicine, Calgary, Canada

## Abstract

**Background:**

The establishment of chronic HBV infection in over 297 million people is due in part through the virus’ highly stable covalently closed circular DNA (cccDNA) persisting in the nuclei of infected hepatocytes. Thus, curing HBV will require direct targeting of the minigenome and detailed structural knowledge of the cccDNA to determine vulnerabilities.

**Purpose:**

Previous studies have demonstrated that human protein Sp1 interacts with the HBV genome at the preCore/Core promoter - a critical interaction for viral replication. We have recently discovered that the Sp1-binding region of HBV pre-Core forms a highly ordered G-quadruplex (G4) secondary structure which presents a novel therapeutic anti-HBV target.

**Method:**

Using phage display technologies, we have identified 11 G-quadruplex binding single domain antibodies that can target the G4 present within the cccDNA. Using recombinant protein expression we characterized the strongest binder (S10) and its interaction with a 22nt HBV pre-Core G-quadruplex forming oligo. Using MicroScale Thermophoresis (MST), the binding affinity (K_D_) between S10 and the target G4 was determined to be ~218 nM, which is 100x stronger for folded G4 versus unfolded oligos of the same sequence. Using oligos of (~60-90%) sequence similarity, it was observed that S10 has a K_D_ for the target G4 that was at least 10x higher than similar sequences. To determine the effect of S10 on HBV replication, we have transduced the HepG2-NTCP-A3 cell line to express these sdAbs and are in the process of evaluating antiviral effects and target specificity for cccDNA.

**Result(s):**

Using biophysical in-vitro approaches, S10 has shown a great potential in being able to discriminate against different G4’s, while having a high degree of affinity as well as high complex stability.

**Conclusion(s):**

The ability of these G4 binding single domain antibodies to discriminate between different sequences or secondary DNA structure provides an insight into how they can be exploited in future HBV therapeutic strategies.

**Disclosure of Interest:**

None Declared

